# Evaluation of Current Antiemetic Therapy Response in Patients Undergoing MEC or HEC Regimens in Portugal

**DOI:** 10.3390/curroncol30020117

**Published:** 2023-01-24

**Authors:** António Araújo, Nuno Tavares, Ana Luísa Faria, Rosa Gomes, Joana Carvalho Mendonça, Bárbara Parente, Andreia Capela, Fernando Barata, Ana Macedo

**Affiliations:** 1 Hospital de Santo António, Centro Hospitalar Universitário do Porto, Largo Prof. Abel Salazar, 4099-001 Porto, Portugal; 2 Centro Hospitalar Universitário de São João, 4200-319 Porto, Portugal; 3 Centro Hospitalar de Entre-o-Douro e Vouga, E.P.E., 4520-211 Santa Maria da Feira, Portugal; 4 Centro Hospitalar de Trás-os-Montes e Alto Douro E.P.E., 5000 508 Vila Real, Portugal; 5 Hospital da Senhora da Oliveira, 4835-044 Guimarães, Portugal; 6 Hospital CUF Porto, 4100-180 Porto, Portugal; 7 Centro Hospitalar de Vila Nova de Gaia/Espinho, 4434-502 Vila Nova de Gaia, Portugal; 8 Associação de Investigação de Cuidados de Suporte em Oncologia (AICSO), 4410-406 Vila Nova de Gaia, Portugal; 9 Centro Hospitalar Universitário de Coimbra, 3049-002 Coimbra, Portugal; 10 Departamento de Ciências Biomédicas e Medicina, Universidade do Algarve, 8005-139 Faro, Portugal

**Keywords:** neoplasms, chemotherapy, nausea, vomiting, CINV

## Abstract

Chemotherapy-induced nausea and vomiting (CINV) negatively impact cancer patients’ quality of life and treatment outcomes. This study evaluated the achievement of complete response to CINV prophylaxis during the first five days after chemotherapy in adult outpatient cancer clinics with solid malignant tumours receiving Moderate or Highly Emetogenic Chemotherapy (MEC or HEC) in Portugal. During the study, patients completed three evaluations, and nausea severity and CINV impact on patients’ daily life was assessed. A complete response (no emetic episodes, no use of rescue antiemetic medication, and no more than mild nausea) was observed in 72% of the cycles (N = 161) throughout the five days after chemotherapy. Amongst the patient population, 25% classified their CINV episodes as severe. Though more than half of the patients achieved a complete response, suggesting that a therapeutic effort is being made to minimise this side effect, the overall scenario is barely optimistic. Significantly, new CINV-control measures in MEC/HEC patients should be adopted, specifically avoiding the single use of dexamethasone and 5-HT3 and raising awareness of using NK1-RAs. Thus, it is critical to improve CINV prophylactic treatment and implement practical international antiemetic guidelines in Portuguese clinical practice, envisaging the improvement of supportive care for cancer patients.

## 1. Introduction

Chemotherapy-induced nausea and vomiting (CINV) is a frequent adverse event that affects up to 60–80% of cancer patients without antiemetic prophylaxis treatment [1]. CINV significantly impairs cancer patients’ quality of life and affects the treatment outcome, including chemotherapy (CT) discontinuation, long-term response to anticancer treatment, and, ultimately, patient survival [1,2,3,4]. As nausea and vomiting are considered a certainty during the CT treatment, they represent a critical concern, being, thus, frequently anticipated by cancer patients [2]. Notably, patient expectations regarding the consequences of the treatment are essential, considering that previous studies on nausea and vomiting suggest an association between the expectancy of CINV and its occurrence during treatment [3]. Furthermore, it has been previously demonstrated that uncontrolled CINV is a pivotal factor for CINV repetition in subsequent cycles, increasing the probability of its occurrence by 6.5 and 14 times in cycles 2 and 3, respectively [5,6]. Therefore, it is crucial to address CINV from a preventive perspective so that patients remain engaged in their CT regimens, improving their compliance with anticancer treatments and their quality of life.

Previous studies described that current antiemetic treatments provide suboptimal CINV control, particularly during the delayed phase in patients receiving Highly Emetogenic Chemotherapy (HEC) or Moderate Emetogenic Chemotherapy (MEC) [7]. Furthermore, a prospective observational study carried out in Portugal that analysed how health care professionals (HCP) perceive CINV compared to the effects directly reported by patients disclosed that HCP significantly underestimates the incidence and the negative impact of acute and delayed nausea in the daily life of patients, after HEC and MEC [8].

Several international scientific societies, such as the Multinational Association for Supportive Care in Cancer (MASCC), the European Society for Medical Oncology (ESMO), and the National Comprehensive Cancer Network (NCCN), offer recommendations for antiemetic prophylaxis according to the emetogenicity grade of the oral antineoplastic agents. Globally, these guidelines share many fundamental similarities and focus on preventing acute and delayed nausea and vomiting induced by HEC and MEC. Notably, a recent update on MASCC/ESMO guidelines included two newly U.S. Food and Drug Administration (FDA) and European Medicines Agency (EMA)-approved neurokinin-1 receptor antagonists (NK1-RAs), whose role in preventing acute and delayed nausea and vomiting is still under discussion [9]. According to the recommendations of the Portuguese Oncology Nursing Association, the antiemetic agents used for the prevention of CINV are 5-HT3 receptor antagonists (dolasetron, granisetron, ondansetron, palonosetron); neurokinin-1 receptor antagonists (aprepitant, fosaprepitant, NEPA (nesupitant plus palonosetron)); corticosteroids (dexamethasone, methylprednisolone); dopamine receptor antagonists (metoclopramide, haloperidol), and benzodiazepines (lorazepam). Importantly, this working group also emphasises that an adequate regimen must be tailored to each situation [10].

Although the international guidelines for the prevention and treatment of CINV are widespread, their application in a real-life context remains low [7], and the adherence of HCP to those guidelines is considered a significant problem [8].

Thus, the present study aims to raise awareness of supportive care for cancer patients, highlighting the need to improve CINV prophylactic treatment and the adoption of guidelines in Portuguese clinical practice. The primary objective of this study was to evaluate the achievement of a complete response to CINV prophylaxis during the first five days after CT in patients receiving HEC or MEC in Portugal. The secondary objectives were to address the impact of CINV on the quality of life in cancer patients receiving HEC or MEC and to describe all emetic episodes seven days after CT.

## 2. Methods

### 2.1. Study Design

This observational, non-interventional, prospective, multicenter study aimed to collect real-life data on CINV control with current antiemetic therapies in patients naïve and non-naïve to MEC or HEC in an outpatient cancer clinic in Portugal.

### 2.2. Study Setting and Patient Population

The study included adult patients (≥18 years) CT-naïve or CT-non-naïve, both males and females, with any solid malignant tumours, with ECOG Scale of Performance Status between 0 and 2. Each patient was assessed once per cycle, not limited to only one cycle evaluation per CT regimen. The CT-non-naïve patients included those treated with single-day HEC or MEC or who had already received at least two CT cycles, considering the current CT regimen. The antiemetic and anticancer therapies were prescribed at the investigator’s discretion, meaning that patient assignment to a therapeutic strategy was not decided in advance by the study protocol but fell within current clinical practice. Patients had to be able to complete written questionnaires and complete patient diaries. Patients with nausea or vomiting not related to any CT cycle, other disorders or medications, or under chemoradiotherapy or CT with low or minimal emetic risk, or vomiting within 24 h (h) before the CT cycle or with unstable concomitant diseases or uncontrolled brain metastasis were excluded from the study. Pregnant or breastfeeding women and patients under chronic systemic corticosteroid treatment were also excluded.

### 2.3. Sample Size Determination

The study was designed to include *ca.* 120 patients with solid malignant tumours treated with single-day HEC or MEC who had already received at least two CT cycles. The sample size was determined based on the study’s primary objective, which aimed to evaluate the percentage of patients with a complete response for 120 h after CT, in patients receiving HEC or MEC and under CINV prophylaxis guidelines, in Portugal. The sampling calculation assumed a complete response (no vomiting, no rescue medication, and no more than mild nausea) in 55% of the CT cycles. Considering this hypothesis, and an error margin lower than 7%, for a 95% interval, it was necessary to evaluate 194 cycles.

### 2.4. Procedures and Assessments

**Figure 1:**
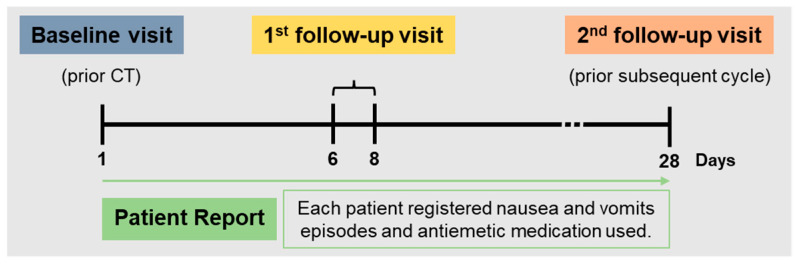
Schematic process of data collected during the study.

The antiemetic and anticancer therapies were prescribed according to current clinical practice. Specifically, the antiemetic agents used for the prevention of CINV were 5-HT3 receptor antagonists (dolasetron, granisetron, ondansetron, palonosetron); neurokinin-1 receptor antagonists (aprepitant, fosaprepitant, NEPA (nesupitant plus palonosetron)); corticosteroids (dexamethasone, methylprednisolone); dopamine receptor antagonists (metoclopramide, haloperidol), and benzodiazepines (lorazepam). No additional diagnostic or monitoring procedures were applied to the patients except if the investigator decided. CT regimens were categorised according to international guidelines (MASCC, ESMO, and NCCN) and based on the emetogenic potential of the agent. Each patient was evaluated three times during the study period: baseline, follow-up 1, and follow-up 2. Baseline visits occurred on day 1, before the administration of CT, and follow-up visits occurred following a clinical practice. The first follow-up visit occurred between days 6 and 8 of the CT cycle, and the second follow-up was on day 28, just before the subsequent CT cycle. During the study period, each patient filled out a diary to record nausea and vomits episodes and antiemetic medication use (Figure 1).

### 2.5. Statistical Analysis

The primary endpoint was the proportion of patients who achieved a complete response to CINV prophylaxis in the first five days (120 h) after CT and the respective 95% confidence interval.

The secondary endpoints were the percentage of change in the FLIE questionnaire score from baseline to day 7, the median and mean time to failure, defined as the time until the CINV control (occurrence of the first emetic episode or the first use of rescue medication), and the characterisation of nausea severity during the five days after CT measured by a Likert scale.

All variables were described using mean, median, standard deviation, interquartile range (IQR), maximum and minimum for continuous variables, and absolute (*n*) and relative frequencies (%) for categorical variables. Time to failure was estimated using Kaplan–Meier analysis and the response agreement between the physician’s assessment and the patient’s self-report through Cohen’s Kappa test. A 0.05 significance level was considered for all tests performed.

## 3. Results

A total of 115 patients were included from eight sites in Portugal. The baseline patients’ demographic and clinical characteristics are disclosed in Table 1.

Most participants were female (61%), and the median age at baseline was 59.5 (IQR: 16; 51–67 CI 95%). The most frequent primary diagnosis was breast cancer (30%), followed by colon cancer (15%) and lung cancer (14%). Most of the participants in the study had an ECOG Performance Status of 0, and only a minority had a history of previous CT or radiation therapy (31% and 18%, respectively). The CT regimens and antiemetic prophylaxis performed among participants are shown in Table 2 and Table 3. The most common CT regimens were based on oxaliplatin or irinotecan, and the most used antiemetic agents were dexamethasone, metoclopramide, and ondansetron.

Regarding the primary endpoint, a complete response (no emetic episodes, no use of rescue antiemetic medication, and no more than mild nausea) was observed in 72% [95% CI 63.8–79.3] of the CT cycles during the 120 h (5 days) after CT. Between days 1 and 7 after CT, patients reported having experienced at least one emetic episode, one nausea episode, or used rescue antiemetic medication in 43% of the cycles. Significantly, this percentage dropped to 29% if considering only emetic episodes or using rescue medication.

The complete characterisation of nausea and emesis episodes and their severity classification are presented in Table 4. The median time until the first emetic episode or first use of rescue antiemetic therapy was 15 h, varying from 8 to 148 h from the first CT administration. Approximately 25% of patients classified their emetic/nausea episodes as severe, 30% as mild [1,2], and the remaining as moderate.

Regarding the assessment of the patient’s quality of life, those without and with CINV have a mean FLIE Total Score of 46.2 at baseline and 46.4 at the first follow-up visit (*p* = 0.502) and a mean FLIE Total Score of 56.1 at baseline and 60.9 at the first follow-up visit (*p*-value = 0.149), respectively. Therefore, there was no difference in FLIE Total Score between baseline and the first follow-up visit (7 days after CT) in patients with and without CINV. FLIE Total Scores are reported in Table 5.

Before the CT administration, the physicians expected that 40% of patients would experience nausea or emetic episodes between days 1 and 7 after the CT cycle. The severity of expected patients’ nausea and emesis episodes for seven days after CT was mainly graded from 1 to 3, according to physicians’ expectations.

According to physicians’ assessment of patients’ diaries analysis, CINV occurred in 37% of patients between days 1 and 7 after the CT cycle. Patients’ nausea and emesis episodes for seven days after CT were mainly classified as a severity grade of 3 or 4. Accordingly to the physicians’ assessment, CINV occurred in 27% of patients between days 8 and 28 after CT and was also classified mainly as grade 3 or 4. In fact, 42% of the patients reported having experienced nausea or emetic episodes in the first week after CT.

The response agreement between physician assessment and patient reports was assessed using Cohen’s Kappa and disclosed 92.7% (*p* < 0.001).

## 4. Discussion

Despite substantial advances in supportive care of cancer patients over the last decades, through developing recommendations to improve antiemetic strategies [9,11,12], CINV remains one of cancer treatments’ most distressing and debilitating adverse effects [13]. Accordingly, this study confirms the high prevalence of CINV as in 43% of all the CT cycles, patients reported having experienced at least one emetic episode, one nausea episode, or used rescue antiemetic medication during the first week after CT.

Though the data herein collected shows that the prophylaxis treatments were overall effective in the prevention of emesis in 72% of the cycles (patients have a complete response, meaning no emetic episodes, no use of rescue antiemetic medication, and no mild nausea for five days after CT), there is still space for improvement. These results disclose that the doublet regimen (specifically, dexamethasone and a 5-HT3) is insufficient to prevent CINV episodes in MEC and HEC patients. Based on this, it is fundamental to discuss new therapeutic strategies comprising the use of NK1-RAs medication and the adoption of more effective international antiemetic guidelines in the Portuguese clinical practice to improve CINV supportive care for cancer patients.

Nevertheless, a complete emetic response is not an appropriate endpoint to characterise the treatment effect on nausea [14], and control of nausea still represents the most critical challenge in CINV. Although nausea and vomiting have always been treated as unified symptoms, they represent different phenomena. Notably, nausea has a higher incidence and is more challenging to evaluate, quantify and control with medication [15,16]. Additionally, our data corroborate that nausea has also been described to affect patients’ quality of life [15,17,18]. Nausea episodes are frequently described during the first week after CT compared to emesis episodes. As already described, the optimal control of CINV is particularly relevant concerning nausea, and its adequate prevention, rather than just vomiting, remains an unmet need in patients submitted to CT [16,19,20].

No significant differences in the patient’s quality of life were detected. Specifically, the variation in the score of the FLIE questionnaire between baseline and the first follow-up visit after seven days upon CT was not significant, which might result from the “ceiling effect” caused by high baseline and follow-up scores that usually lead to modest alterations.

Noteworthy, our study disclosed a high response agreement between patients’ reports and physicians’ assessments concerning nausea or emetic episodes. This finding differs from previous studies on this topic, where HCP significantly underestimate the incidence of acute and delayed nausea after HEC and MEC compared to patients’ reports [7,21]. This may indicate that, in the last decades, physicians are becoming increasingly aware of CINV, suggesting the achievement of an important milestone in implementing CINV prophylaxis strategies [22,23].

## 5. Conclusions

In conclusion, this study discloses that a significant proportion of complete response has been achieved, suggesting that a therapeutic effort is being made to minimise this unagreeable side effect of anticancer regimens and improve cancer patients’ quality of life. Nonetheless, despite the several improvements in clinical practice and the discovery of new treatments, CINV still represents a pertinent question in cancer patients submitted to HEC or MEC in Portugal, pointing out the need to implement more effective strategies and adopt antiemetic guideline directives.

## Figures and Tables

**Table 1:** Demographic and clinical characterisation of the patients.

Sociodemographic and Clinical Characteristics (*n* = 115)		*n*
**Sex**		
Female		70 (61%)
Male		45 (39%)
**Age at baseline (years)**		
Mean (SD)		57.9 (12.4)
Median (IQR)		59.5 (51–67)
**Employment status**		
Employed		41 (35.7%)
Retired		37 (32.2%)
Unemployed		8 (7.0%)
Domestic		7 (6.1%)
Missing		22 (19.1%)
**Primary Cancer Diagnosis**		
Breast Cancer		33 (28.7%)
Colon Cancer		16 (13.9%)
Lung Cancer		15 (13.0%)
Pancreas Cancer		14 (12.2%)
Gastric Cancer		11 (9.6.%)
Other		18 (15.7%)
ND/NK		2 (1.7%)
Missing		5 (4.3%)
**ECOG Performance Status**		
0		69 (60.0%)
1		43 (37.4%)
2		2 (1.7%)
Missing		1 (0.9%)
**Prior radiation therapy**		
Yes		21 (18.2%)
No		89 (77.4%)
ND/NK		4 (3.5%)
Missing		1 (0.9%)
**Prior chemotherapy treatment**		
Yes		35 (30.4%)
No		75 (65.2%)
ND/NK		4 (3.5%)
Missing		1 (0.9%)
**Type of previous chemotherapy**		
Oral Antineoplastic Agents	Moderate to High	3
	Minimal to Low	7
Intravenous Antineoplastic Agents	High	6
	Moderate	28
	Low	22
	Minimal	8
**Missing**		1

IQR: Interquartile range; ND/NK: Not defined/Not Known; SD: Standard deviation.

**Table 2:** Current chemotherapy regimens.

Chemotherapy Regimens (*n* = 161)	*n* (%)
**Oral agents**	
Temozolomide > 75 mg/m^2^/d	2 (1.2)
E toposide	2 (1.2)
Cyclophosphamide ≥ 100 mg/m^2^/d	1 (0.6)
**IV Agents-High emetic risk (HEC)**	
Anthracyclin and cyclophosphamide combination	23 (14.3)
Carboplatin AUC ≥ 4	20 (12.4)
Cisplatin	6 (3.7)
Epirubicin > 90 mg/m^2^	3 (1.9)
Cyclophosphamide > 1500 mg/m^2^	1 (0.6)
Ifosfamide ≥ 2 mg/m^2^	1 (0.6)
Doxorubicin ≥ 60 mg/m^2^	1 (0.6)
**IV Agents-Moderate emetic risk (MEC)**	
Oxaliplatin	65 (40.4)
Irinotecan	34 (21.1)
Cyclophosphamide ≤ 1500 mg/m^2^	9 (5.6)
Carboplatin AUC < 4	9 (5.6)
Ifosfamide ≥ 60 mg/m^2^	1 (0.6)
**Doxorubicin < 60 mg/m^2^**	**1 (0.6)**

IV: Intravenous.

**Table 3:** Current antiemetic prophylaxis/medication.

Antiemetic Prophylaxis/Medication (*n* = 161)	%
**Serotonin Agonists**	
Ondansetron	28
Palonosetron	13
**Neurokinin-1 Antagonist**	
Aprepitant	2
Fosaprepitant	7
Netupitant + Palonosetron	10
**Adrenocortical Steroid**	
Dexamethasone	50
** Atypical Antipsychotic**	
Olanzapine	1
**Benzodiazepine**	
Lorazepam	1
**Other**	
**Metoclopramide**	**41**

**Table 4:** Nausea and emesis episodes characterisation.

Day after CT	Nausea Episodes (*n* = 161)		Emetic Episodes (*n* = 161)		Use of Rescue Antiemetic Medication (*n* = 161)
	%	Median (range)	%	Median (range)	%
1	24.8	2.5 (1–12)	7.9	3 (2–5)	77.4
2	27.8	3 (1–10)	9.5	3 (2–4)	80.0
3	30.2	3 (1–10)	12.9	2 (1–4)	71.1
4	28.2	2 (1–10)	14.5	3 (1–5)	75.0
5	28.6	2 (1–10)	11.1	2.5 (1–4)	64.9
6	26.2	3 (1–6)	8.8	2 (1–3)	62.9
7	25.4	2 (1–10)	6.4	2.5 (1–3)	59.4

CT: Chemotherapy.

**Table 5:** Quality of Life assessment based on FLIE questionnaire score.

FLIE Total Score (*n* = 161)		Baseline (*n* = 161)	First Follow-up (*n* = 161)
**Patients without CINV**	Mean (SD)	46.2 (11.8)	46.4 (9.2)
	Median	48	48
	Range (min-max)	70 (18–88)	53 (18–71)
**Patients with CINV**	Mean (SD)	56.1 (11.3)	60.9 (15.3)
	Median	56	62
	Range (min-max)	44 (35–79)	68 (27–95)

CINV: Chemotherapy-induced nausea and vomiting; FLIE: Functional Living Index-Emesis questionnaire; SD: Standard deviation.

## Data Availability

The data presented in this study are available on request from the corresponding author.
